# County-Level Maternal Vulnerability and Preterm Birth in the US

**DOI:** 10.1001/jamanetworkopen.2023.15306

**Published:** 2023-05-25

**Authors:** Elizabeth G. Salazar, Diana Montoya-Williams, Molly Passarella, Carolyn McGann, Kathryn Paul, Daria Murosko, Michelle-Marie Peña, Robin Ortiz, Heather H. Burris, Scott A. Lorch, Sara C. Handley

**Affiliations:** 1 Division of Neonatology, The Children’s Hospital of Philadelphia, Philadelphia, Pennsylvania; 2 Leonard Davis Institute of Health Economics, Philadelphia, Pennsylvania; 3 Department of Pediatrics, Perelman School of Medicine, University of Pennsylvania, Philadelphia, Pennsylvania; 4 Emory University School of Medicine and Children’s Healthcare of Atlanta, Atlanta, Georgia; 5 Department of Pediatrics, Institute for Excellence in Health Equity, NYU Grossman School of Medicine, New York, New York; 6 Department of Population Health, Institute for Excellence in Health Equity, NYU Grossman School of Medicine, New York, New York

## Abstract

**Importance:**

Appreciation for the effects of neighborhood conditions and community factors on perinatal health is increasing. However, community-level indices specific to maternal health and associations with preterm birth (PTB) have not been assessed.

**Objective:**

To examine the association of the Maternal Vulnerability Index (MVI), a novel county-level index designed to quantify maternal vulnerability to adverse health outcomes, with PTB.

**Design, Setting, and Participants:**

This retrospective cohort study used US Vital Statistics data from January 1 to December 31, 2018. Participants included 3 659 099 singleton births at 22 plus 0/7 to 44 plus 6/7 weeks of gestation born in the US. Analyses were conducted from December 1, 2021, through March 31, 2023.

**Exposure:**

The MVI, a composite measure of 43 area-level indicators, categorized into 6 themes reflecting physical, social, and health care landscapes. Overall MVI and theme were stratified by quintile (very low to very high) by maternal county of residence.

**Main Outcomes and Measures:**

The primary outcome was PTB (gestational age <37 weeks). Secondary outcomes were PTB categories: extreme (gestational age ≤28 weeks), very (gestational age 29-31 weeks), moderate (gestational age 32-33 weeks), and late (gestational age 34-36 weeks). Multivariable logistic regression quantified associations of MVI, overall and by theme, with PTB, overall and by PTB category.

**Results:**

Among 3 659 099 births, 298 847 (8.2%) were preterm (male, 51.1%; female, 48.9%). Maternal race and ethnicity included 0.8% American Indian or Alaska Native, 6.8% Asian or Pacific Islander, 23.6% Hispanic, 14.5% non-Hispanic Black, 52.1% non-Hispanic White, and 2.2% with more than 1 race. Compared with full-term births, MVI was higher for PTBs across all themes. Very high MVI was associated with increased PTB in unadjusted (odds ratio [OR], 1.50 [95% CI, 1.45-1.56]) and adjusted (OR, 1.07 [95% CI, 1.01-1.13]) analyses. In adjusted analyses of PTB categories, MVI had the largest association with extreme PTB (adjusted OR, 1.18 [95% CI, 1.07-1.29]). Higher MVI in the themes of physical health, mental health and substance abuse, and general health care remained associated with PTB overall in adjusted models. While the physical health and socioeconomic determinant themes were associated with extreme PTB, physical health, mental health and substance abuse, and general health care themes were associated with late PTB.

**Conclusions and Relevance:**

The findings of this cohort study suggest that MVI was associated with PTB even after adjustment for individual-level confounders. The MVI is a useful measure for county-level PTB risk that may have policy implications for counties working to lower preterm rates and improve perinatal outcomes.

## Key Points

**Question:**

Is the Maternal Vulnerability Index (MVI), a novel county-level index designed to quantify maternal vulnerability to adverse health outcomes, associated with preterm birth (PTB)?

**Findings:**

In this cohort study using 2018 national birth certificate data for 3 659 099 births, PTB rates were significantly lower among individuals with very low MVI compared with those with very high MVI (7% vs 10%). The association between very high MVI and PTB remained after adjustment for patient characteristics.

**Meaning:**

The findings of this study suggest that the MVI is a useful measure for county-level PTB risk and may provide guidance for communities working to improve perinatal health.

## Teaser

This cohort study examines the association of the Maternal Vulnerability Index (MVI) with preterm birth (PTB) and severity of PTB, both overall and stratified by physical, social, and health care factors included in the index.

## Introduction

Preterm birth (PTB; gestational age <37 weeks) affects 1 in 10 births in the US and is associated with birthing parent and infant mortality and morbidity.^1,2,3,4,5,6,7^ Preterm birth is multifactorial, as highlighted by the myriad of PTB risk factors in the literature, and heterogenous, as evident in the different causes of spontaneous and medically indicated PTB.^8,9,10,11^ Historically, PTB studies focused on maternal characteristics, such as age, obstetric history, preexisting comorbidities, and pregnancy-associated conditions.^8,9,11^ There is increasing appreciation of how neighborhood conditions and community factors influence PTB.^12,13,14,15,16,17^ Poverty,^18^ air pollution,^19,20^ and composite measures of neighborhood quality^21^ are associated with PTB. Communities reflect a complex amalgamation of risk and protective factors. Some studies have used composite indices such as the Centers for Disease Control and Prevention’s Social Vulnerability Index (SVI)^22^ to assess how community factors influence health outcomes, including PTB.^23,24,25,26^ However, neighborhood or community-level indices specific to maternal health and PTB have not been examined.

Modeled after the SVI, the Maternal Vulnerability Index (MVI) was developed to identify where and why US birthing parents experience adverse health outcomes.^27^ Made publicly available in October 2021, this open-source tool provides a county-level index encompassing 43 indicators, categorized into 6 themes reflecting physical, social, and health care landscapes.^27^ Preliminary data show an association between higher MVI and maternal mortality.^27^ The extent to which the MVI affects infant health is unknown. Since PTB is a pregnancy outcome with significant implications for infant health, the objective of this study was to examine the association of the MVI with PTB and severity of PTB, both overall and stratified by MVI theme.

## Methods

### Study Design and Population

We performed a retrospective, cross-sectional study of a deidentified cohort of births using US National Vital Statistics birth certificate data from January 1 through December 31, 2018. This year was chosen given concurrence with MVI data sources, for which most data reflect variables from 2016 to 2021. Eligible births were between 22 plus 0/7 and 44 plus 6/7 weeks of gestation, consistent with American College of Obstetricians and Gynecologists recommendations for neonatal resuscitation.^28^ Birth certificate data were linked to the MVI data using the county Federal Information Processing System (FIPS) code reflecting the maternal county of residence (9398 [0.26%] dropped due to missing linkage data). Births were excluded if they were outside the gestational age range (n = 6509), multiples (n = 126 528), or born outside the US (n = 9398).^29^ This study followed the Strengthening the Reporting of Observational Studies in Epidemiology (STROBE) reporting guideline. The study was reviewed by the Children’s Hospital of Philadelphia Institutional Review Board and deemed not to meet criteria for human participants research and therefore exempt from informed consent.

### Exposure Definitions

The primary exposure was the MVI, a novel index designed to quantify area-level indicators of maternal vulnerability to adverse maternal health outcomes.^27^ Developed by Surgo Ventures, a privately funded group, the MVI was based on a literature review of publications (reports, working papers, books, scientific manuscripts, and review articles) from 2000 through 2020 identifying factors associated with US maternal mortality and morbidity.^27^ Factors with consistent associations from publicly available data sets were included, with review by subject matter experts. Per the Surgo Venture website, further measure development details are being prepared for publication.^27^ The resulting MVI is a composite measure of 43 county-level indicators categorized into 6 themes: (1) reproductive health care; (2) physical health; (3) mental health and substance abuse; (4) general health care; (5) socioeconomic determinants; and (6) physical environment (eTable 1 in Supplement 1).^27^ The MVI score is standardized from 0 to 100 nationally, with 0 being the best score (lowest vulnerability) and 100 the worst (highest vulnerability) and then divided into quintiles of very low (0-20), low (20-40), moderate (40-60), high (60-80), and very high (80-100).^27^ We examined the exposure of MVI quintile overall and by theme.

### Outcomes

The primary outcome was PTB (gestational age <37 weeks). Secondary outcomes were PTB gestational age categories: extreme (<28 weeks), very (28 plus 0/7 to 31 plus 6/7 weeks), moderate (32 plus 0/7 to 33 plus 6/7 weeks), and late (34 plus 0/7 to 36 plus 6/7 weeks).^30^

### Covariates

Maternal variables included in the analysis were based on prior literature of PTB risk factors. These included age (<20, 20-24, 25-34, and ≥35 years)^31^; race and ethnicity (American Indian or Alaska Native, Asian or Pacific Islander, Hispanic, non-Hispanic Black, non-Hispanic White, and >1 race or ethnicity) reported by birth parents on the birth certificate, which was included given the influence of structural racism on perinatal outcomes^32^; insurance type (Medicaid, private, self-pay, other, or missing)^33^; level of educational attainment (<8th grade, 8th-12th grade or no degree, high school or General Educational Development, some college, bachelor’s degree or higher, or missing)^34^; Kotelchuk index (an adequacy of prenatal care utilization index as inadequate, intermediate, adequate, adequate plus, or missing)^5^; body mass index (calculated as weight in kilograms divided by height in meters squared]; categorized as <18.5, 18.5-24.9, 25.0-29.9, ≥30, or missing)^35^; nulliparity (binary variable)^36^; preexisting hypertension (binary variable)^37^; smoking during pregnancy (binary variable)^38^; rurality, which was determined using Urban Influence Codes (UICs) and categorized as metropolitan (UIC 1 or 2), micropolitan (UICs 3, 5, or 8), and noncore (rural; UICs 4, 6, 7, 9, 10, 11, or 12)^39,40^; and birth state.^41^

### Statistical Analysis

Descriptive statistics were reported using counts and percentages for categorical variables and means and SDs for continuous variables. They were calculated using χ^2^ tests for dichotomous variables and analysis of variance for continuous variables. We examined the association of overall MVI quintiles with PTB using multivariable logistic regression with adjustment for demographics and preexisting conditions, clustered by county with robust standard errors and with birth state as a fixed effect. Models were repeated, first examining associations of each of the 6 MVI themes with PTB and then MVI themes with gestational age categories, the latter using multivariate, multinomial logistic regression. The output from the Stata mlogit model is expressed as a ratio of relative risk but for simplicity is reported as an odds ratio (OR).^42,43^ A missing category indicator was created for the insurance type, level of educational attainment, Kotelchuk index, and body mass index, which was integrated into multivariate models. Dedicated analyses of racial disparities in the association between MVI and PTB were outside this study’s scope. However, given the potential collinearity of MVI indicators with measures of structural racism, we conducted a sensitivity analysis without race and ethnicity as a model covariate to decrease the possibility of overadjustment for a significant causal contributing pathway between MVI and the risk of PTB. Statistical analyses were conducted from December 1, 2021, through March 31, 2023, using Stata, version 16 (StataCorp LLC); *P* < .05 was considered statistically significant.

## Results

The analytic cohort included 3 659 099 singleton births at gestational ages from 22 plus 0/7 to 44 plus 6/7 weeks, of which  298 847 (8.2%) were PTBs (0.5% extreme, 0.7% very, 0.9% moderate, and 6.1% late). Among this cohort, 51.1% were male and 48.9% were female. Maternal race and ethnicity included 0.8% American Indian or Alaska Native, 6.8% Asian or Pacific Islander, 23.6% Hispanic, 14.5% non-Hispanic Black, 52.1% non-Hispanic White, and 2.2% more than 1 race or ethnicity. In bivariate analyses, several covariates were associated with higher MVI, including non-Hispanic Black race (7.4% for very low vs 22.6% for very high), American Indian or Alaska Native race (0.4% for very low vs 2.5% for very high), Medicaid (30.4% for very low vs 58.2% for very high), having obesity (23.3% for very low vs 34.7% for very high), smoking during pregnancy (4.4% for very low vs 10.2% for very high), preexisting hypertension (1.6% for very low vs 2.8% for very high), educational attainment of a bachelor’s degree or higher (44.9% for very low vs 16.6% for very high), inadequate prenatal care (5.6% for very low vs 10.2% for very high), and maternal age younger than 20 years (2.7% for very low vs 8.6% for very high) (*P* < .001 for all) (Table). Consistent with prior literature, each of these covariates was associated with PTB in bivariate analyses (eTable 2 in Supplement 1).^31,32,33,34,35,37,38,44^ Preterm birth was more common among individuals who were 35 years old or older, resided in the South US Census region, and resided in micropolitan and noncore (rural) areas (eTable 2 in Supplement 1).

**Table.** Perinatal Characteristics of Births in the US in 2018 by Quintile of MVI

Characteristic	MVI quintile (N = 3 659 099)^a^				
	Very low (n = 980 320)	Low (n = 863 469)	Moderate (n = 732 140)	High (n = 774 985)	Very high (n = 308 185)
All PTBs	68 940 (7.0)	66 322 (7.7)	60 899 (8.3)	71 257 (9.2)	31 429 (10.2)
Gestational age subgroup^b^					
Extreme PTB	3376 (0.3)	3681 (0.4)	3524 (0.5)	4321 (0.6)	1817 (0.6)
Very PTB	5707 (0.6)	5756 (0.7)	5276 (0.7)	6243 (0.8)	2746 (0.9)
Moderate PTB	7183 (0.7)	7080 (0.8)	6742 (0.9)	7852 (1.0)	3384 (1.1)
Late PTB	52 674 (5.4)	49 805 (5.8)	45 357 (6.2)	52 841 (6.8)	23 482 (7.6)
Full-term	911 380 (93.0)	797 147 (92.3)	671 241 (91.7)	703 728 (90.8)	276 756 (89.8)
Birth weight, g, mean (SD)	3338 (544)	3307 (551)	3290 (561)	3261 (568)	3232 (571)
Infant sex					
Female	478 523 (48.8)	422 093 (48.9)	358 049 (48.9)	378 654 (48.9)	150 533 (48.8)
Male	501 797 (51.2)	441 376 (51.1)	374 091 (51.1)	396 331 (51.1)	157 652 (51.2)
Maternal race and ethnicity					
American Indian or Alaska Native	4029 (0.4)	5272 (0.6)	6227 (0.9)	5465 (0.7)	7613 (2.5)
Asian or Pacific Islander	115 518 (11.8)	71 846 (8.3)	29 212 (4.0)	28 440 (3.7)	3925 (1.3)
Hispanic	195 809 (20.0)	226 815 (26.3)	180 934 (24.7)	194 725 (25.1)	64 617 (21.0)
Non-Hispanic Black	72 860 (7.4)	102 427 (11.9)	118 310 (16.2)	168 571 (21.8)	69 632 (22.6)
Non-Hispanic White	567 195 (57.9)	437 027 (50.6)	382 193 (52.2)	361 693 (46.7)	157 581 (51.1)
>1 Race or ethnicity	24 909 (2.5)	20 082 (2.3)	15 264 (2.1)	16 091 (2.1)	4817 (1.6)
Maternal age, y					
<20	26 829 (2.7)	35 500 (4.1)	39 799 (5.4)	49 356 (6.4)	26 629 (8.6)
20-24	129 723 (13.2)	152 205 (17.6)	155 161 (21.2)	180 974 (23.4)	89 822 (29.1)
25-34	597 562 (61.0)	504 225 (58.4)	419 283 (57.3)	432 007 (55.7)	159 114 (51.6)
≥35	226 206 (23.1)	171 539 (19.9)	117 897 (16.1)	112 648 (14.5)	32 620 (10.6)
Maternal insurance					
Private	608 769 (62.1)	449 769 (52.1)	331 531 (45.3)	310 243 (40.0)	99 081 (32.1)
Medicaid	297 772 (30.4)	349 799 (40.5)	336 062 (45.9)	382 324 (49.3)	179 257 (58.2)
Self-pay	30 866 (3.1)	32 867 (3.8)	34 879 (4.8)	40 507 (5.2)	13 647 (4.4)
Other	38 376 (3.9)	26 019 (3.0)	24 970 (3.4)	35 955 (4.6)	15 308 (5.0)
Missing	4537 (0.5)	5015 (0.6)	4698 (0.6)	5956 (0.8)	892 (0.3)
Maternal educational attainment					
Grade 8 or less	27 144 (2.8)	26 425 (3.1)	25 804 (3.5)	25 122 (3.2)	10 401 (3.4)
Grades 9-12, no degree	60 485 (6.2)	76 342 (8.8)	75 148 (10.3)	93 069 (12.0)	42 793 (13.9)
High school or GED	181 873 (18.6)	211 489 (24.5)	204 792 (28.0)	231 982 (29.9)	106 702 (34.6)
Some college	249 614 (25.5)	240 392 (27.8)	212 855 (29.1)	228 418 (29.5)	96 434 (31.3)
Bachelor’s degree or higher	439 796 (44.9)	295 355 (34.2)	207 816 (28.4)	192 012 (25.8)	51 039 (16.6)
Missing	21 408 (2.2)	13 466 (1.6)	5725 (0.8)	4382 (0.6)	816 (0.3)
Kotelchuck index^c^					
Inadequate	55 011 (5.6)	63 852 (7.4)	69 339 (9.5)	85 772 (11.1)	31 361 (10.2)
Intermediate	244 238 (24.9)	212 543 (24.6)	174 888 (23.9)	197 272 (25.5)	76 740 (24.9)
Adequate	488 362 (49.8)	411 550 (47.7)	340 903 (46.6)	329 555 (42.5)	138 056 (44.8)
Adequate plus	171 350 (17.5)	153 874 (17.8)	130 086 (17.8)	137 829 (17.8)	56 827 (18.4)
Missing	21 359 (2.2)	21 650 (2.5)	16 924 (2.3)	24 557 (3.2)	5201 (1.7)
Nulliparous	390 818 (39.9)	334 513 (38.7)	276 184 (37.7)	291 694 (37.6)	109 538 (35.5)
Maternal BMI					
Underweight (<18.5)	28 167 (2.9)	27 549 (3.2)	23 099 (3.2)	25 721 (3.3)	10 270 (3.3)
Normal (18.5-24.9)	446 155 (45.5)	361 562 (41.9)	291 512 (39.8)	305 986 (39.5)	107 440 (34.9)
Overweight (25.0-29.9)	257 358 (26.3)	223 746 (25.9)	192 496 (26.3)	199 105 (25.7)	79 043 (25.6)
Obesity (≥30.0)	228 058 (23.3)	226 819 (26.3)	209 296 (28.6)	227 820 (29.4)	106 813 (34.7)
Missing	20 582 (2.1)	23 793 (2.8)	15 737 (2.1)	16 353 (2.1)	4619 (1.5)
Any smoking during pregnancy	43 343 (4.4)	48 097 (5.6)	56 131 (7.7)	57 785 (7.5)	31 419 (10.2)
Preexisting hypertension	16 049 (1.6)	15 780 (1.8)	15 637 (2.1)	18 081 (2.3)	8634 (2.8)
Gestational hypertension	62 717 (6.4)	56 416 (6.5)	53 527 (7.3)	57 060 (7.4)	24 382 (7.9)
Gestational diabetes	73 696 (7.5)	56 489 (6.5)	49 084 (6.7)	44 098 (5.7)	17 440 (5.7)
Rurality^d^					
Metropolitan	904 187 (92.2)	778 835 (90.2)	630 144 (86.1)	674 711 (87.1)	175 201 (56.8)
Micropolitan	47 071 (4.8)	52 541 (6.1)	67 470 (9.2)	63 505 (8.2)	67 210 (21.8)
Noncore	29 062 (3.0)	32 093 (3.7)	34 526 (4.7)	36 769 (4.7)	65 774 (21.3)
US Census region					
Northeast	278 428 (28.4)	217 817 (25.2)	66 454 (9.1)	22 236 (2.9)	16 (0.01)
Midwest	231 458 (23.6)	191 293 (22.2)	179 417 (24.5)	148 539 (19.2)	18 300 (5.9)
South	109 858 (11.2)	134 389 (15.6)	346 101 (47.3)	559 075 (72.1)	285 931 (92.8)
West	360 576 (36.8)	319 970 (37.1)	140 168 (19.1)	45 135 (5.8)	3938 (1.3)

Abbreviations: BMI, body mass index (calculated as weight in kilograms divided by height in meters squared); GED, General Educational Development; MVI, Maternal Vulnerability Index; PTB, preterm birth.

^a^ The MVI scores range from 0 (lowest vulnerability) to 100 (highest vulnerability). Unless otherwise indicated, data are expressed as No. (%) of births. Percentages have been rounded and may not total 100. There were no missing variables for gestational age, as it was used to define the cohort. Missing variables are otherwise indicated. All variables had associated *P* < .001.

^b^ Preterm birth indicates gestational age less than 37 weeks; extreme PTB, less than 28 weeks; very PTB, 28 to less than 32 weeks; moderate PTB, 32 to less than 34 weeks; late PTB, 34 to less than 37 weeks; and full-term, at least 37 weeks.

^c^ The Kotelchuck index, or Adequacy of Prenatal Care Utilization Index, describes the adequacy of received prenatal care using birth certificate data. Inadequate indicates less than 50% of expected visits; intermediate, 50% to 79% of expected visits; adequate, 80% to 109% of expected visits; and adequate plus, 110% or more of expected visits.

^d^ Rurality is defined using Urban Influence Codes (UICs), with UIC of 1 or 2 indicating metropolitan; UIC 3, 5, or 8, micropolitan; and UIC 4, 6, 7, 9, 10, 11, or 12, noncore.

### PTB Overall

**Figure 1.**
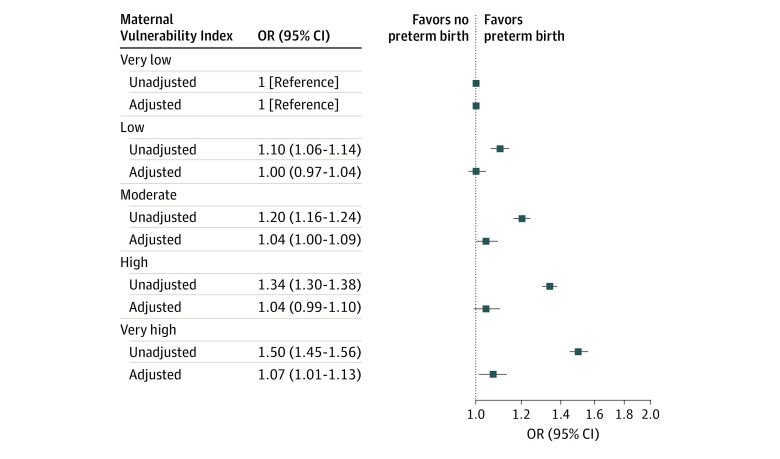
Unadjusted and Adjusted Associations of Maternal Vulnerability Index (MVI) With Preterm Birth by Gestational Age Categories Compared With Full-term Births Associations are reported as odds ratios (ORs). The MVI scores range from 0 (lowest vulnerability) to 100 (highest vulnerability). Where indicated, ORs are adjusted for age, race and ethnicity, insurance, educational attainment, Kotelchuk index of adequate prenatal care, body mass index, nulliparity, preexisting hypertension, smoking during pregnancy, rurality, infant sex, and US Census region.

Preterm birth rates were lower among individuals with very low MVI compared with those with very high MVI (7.0% vs 10.2%; *P* < .001) (Table). Compared with very low MVI, the 4 higher MVI quintiles were associated with PTB in the unadjusted analysis (OR, 1.50 [95% CI, 1.45-1.56]) (Figure 1). The association with very high MVI and PTB persisted in the adjusted analysis and was associated with 7% greater adjusted odds of PTB (Figure 1) (adjusted OR, 1.07 [95% CI, 1.01-1.13]).

**Figure 2.**
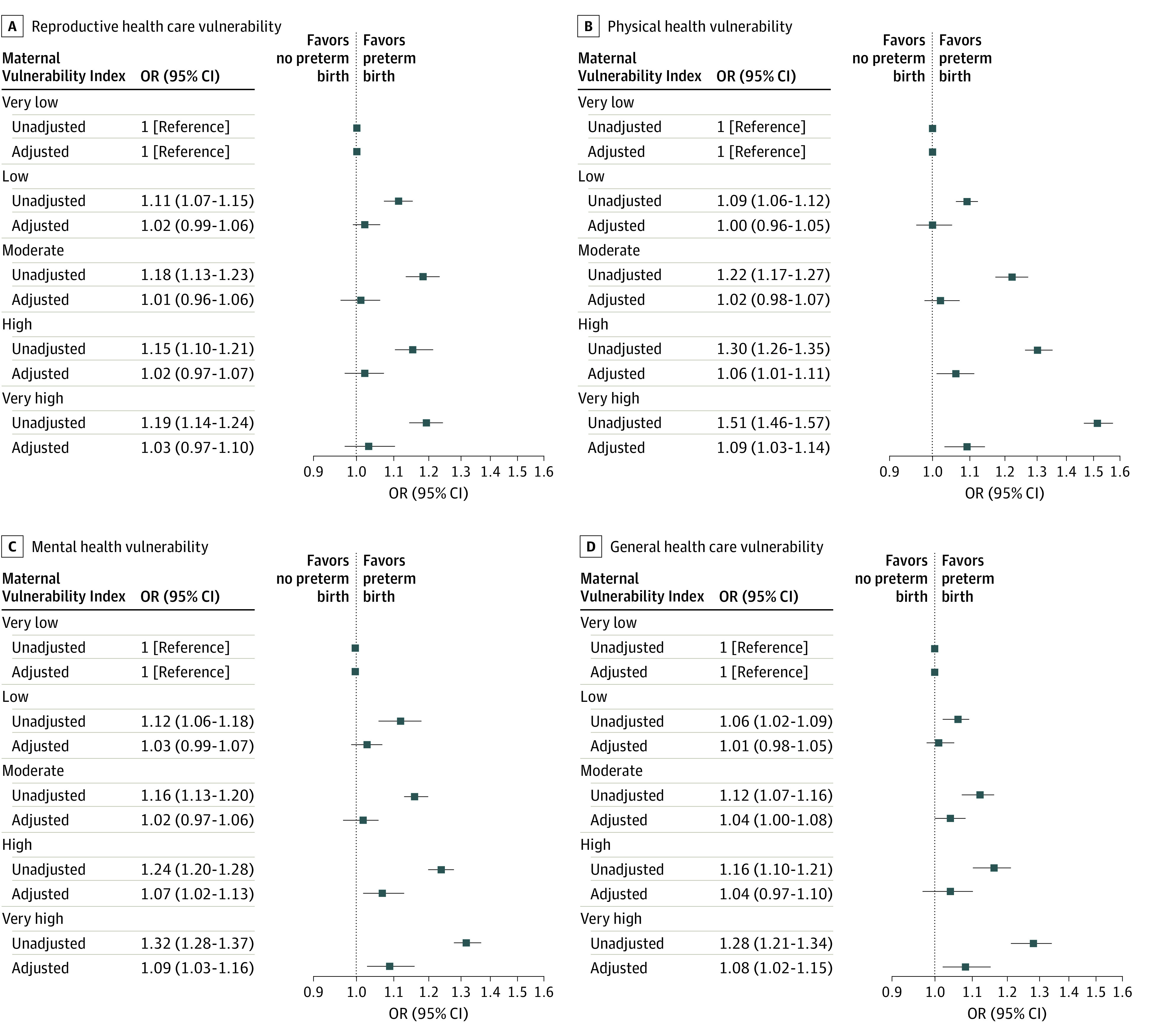
Unadjusted and Adjusted Associations of Maternal Vulnerability Index (MVI) Themes of Reproductive Health Care, Physical Health, Mental Health and Substance Abuse, and General Health Care, With Preterm Births Compared With Full-term Births Preterm birth indicates gestational age younger than 37 weeks. Associations are reported as odds ratios (ORs). The MVI scores range from 0 (lowest vulnerability) to 100 (highest vulnerability). Where indicated, ORs are adjusted for age, race and ethnicity, insurance, educational attainment, Kotelchuk index of adequate prenatal care, body mass index, nulliparity, preexisting hypertension, smoking during pregnancy, rurality, infant sex, and US Census region.

**Figure 3.**
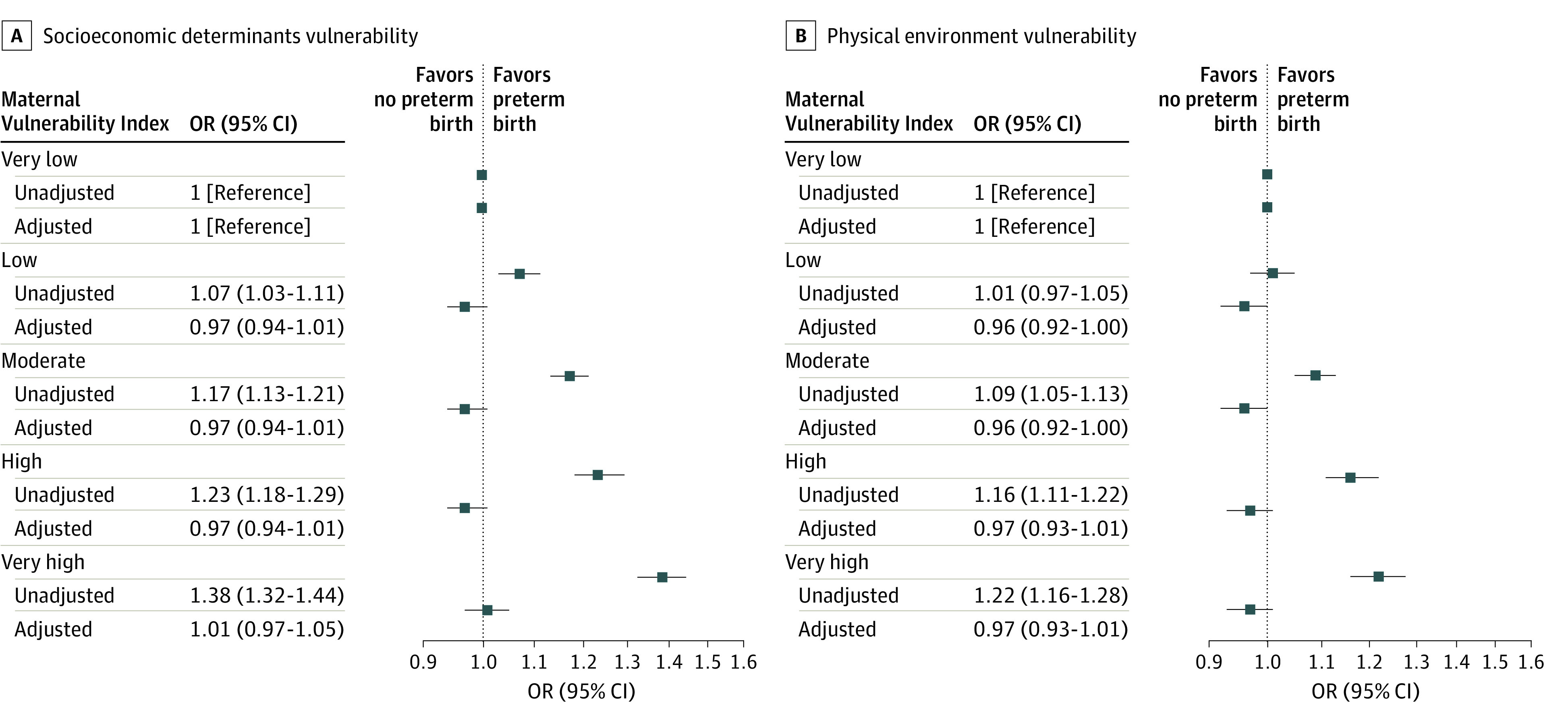
Unadjusted and Adjusted Associations of Maternal Vulnerability Index (MVI) Themes of Socioeconomic Determinants and Physical Environment With Preterm Births Compared With Full-term Births Preterm birth indicates gestational age younger than 37 weeks. Associations are reported as odds ratios (ORs). The MVI scores range from 0 (lowest vulnerability) to 100 (highest vulnerability). Where indicated, ORs are adjusted for age, race and ethnicity, insurance, educational attainment, Kotelchuk index of adequate prenatal care, body mass index, nulliparity, preexisting hypertension, smoking during pregnancy, rurality, infant sex, and US Census region.

We then examined the association between each MVI theme and PTB. Across the 6 themes, PTB rates were lower among individuals who had very low MVI relative to those with very high MVI (eTable 3 in Supplement 1). This was most pronounced in the PTB rates for the MVI themes of physical health (7.0% among those with very low MVI vs 10.2% among those with very high MVI) and socioeconomic determinants (7.0% among those with very low MVI vs 9.5% among those with very high MVI). Each MVI theme was associated with PTB in unadjusted analyses (Figure 2 and Figure 3). In adjusted analyses, very high MVI in the themes of physical health (adjusted OR, 1.09 [95% CI, 1.03-1.14]), mental health and substance abuse (adjusted OR, 1.09 [95% CI, 1.03-1.16]), and general health care (adjusted OR, 1.08 [95% CI, 1.02-1.15]) remained associated with PTB (Figure 2). As the MVI quintile dropped from very high to high, associations were attenuated.

### PTB Gestational Age Categories

**Figure 4.**
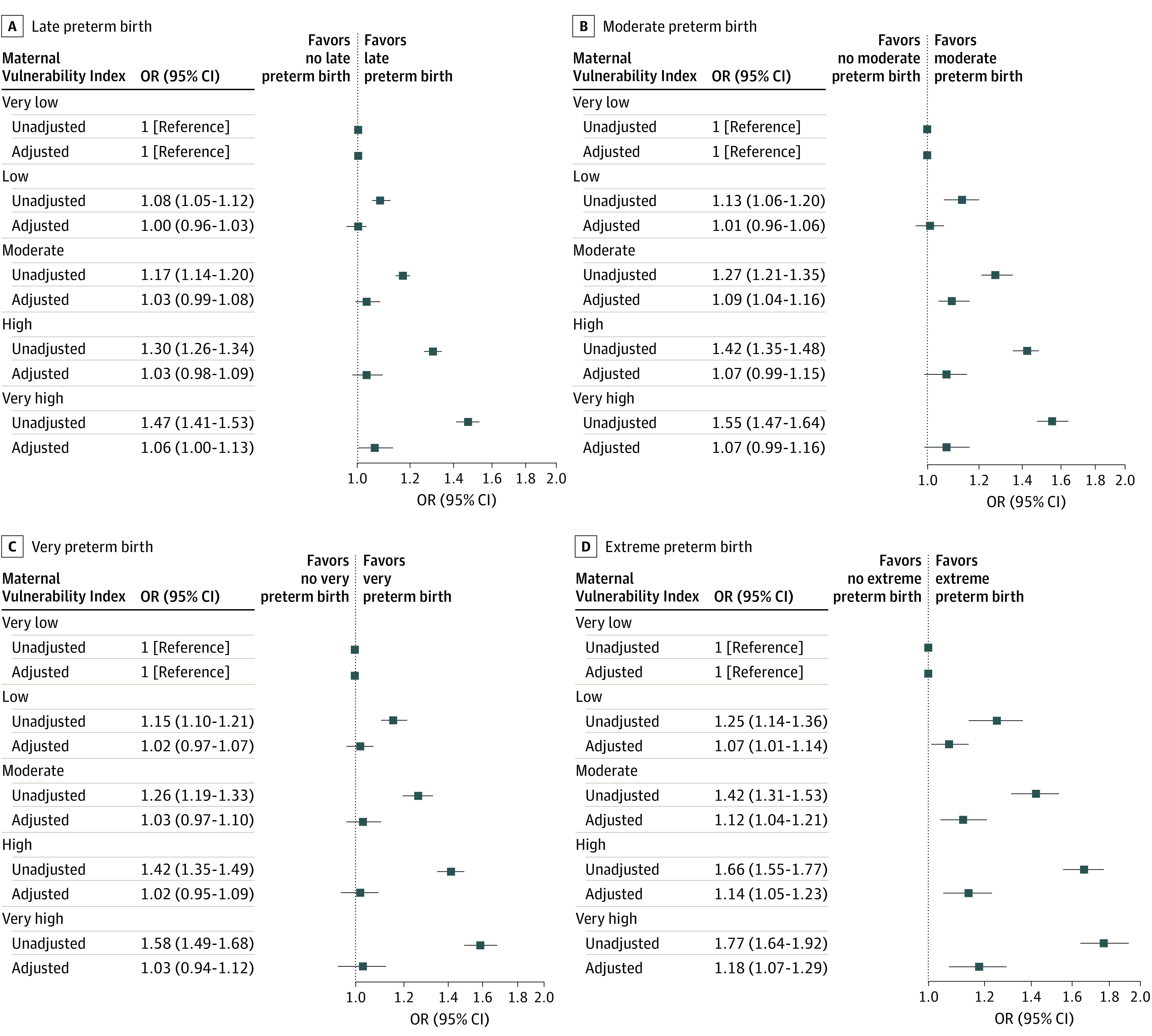
Unadjusted and Adjusted Associations of Maternal Vulnerability Index (MVI) With Preterm Births by Gestational Age Categories Compared With Full-term Births Associations are reported as odds ratios (ORs). The MVI scores range from 0 (lowest vulnerability) to 100 (highest vulnerability). Where indicated, ORs are adjusted for age, race and ethnicity, insurance, educational attainment, Kotelchuk index of adequate prenatal care, body mass index, nulliparity, preexisting hypertension, smoking during pregnancy, rurality, infant sex, and US Census region. Late preterm birth indicates 34 to less than 37 weeks; moderate preterm birth, 32 to less than 34 weeks; very preterm birth, 28 to less than 32 weeks; extreme preterm birth, less than 28 weeks.

When examining associations of the MVI with gestational age categories, higher MVI was associated with greater odds of PTB in all gestational age categories in unadjusted analyses (Figure 4). In adjusted analyses, the point estimates for higher MVI were largest with respect to extreme PTB (Figure 4). Compared with very low MVI, each increasing MVI quintile (low, moderate, high, and very high) was associated with greater odds of extreme PTB, with very high MVI being associated with 18% greater adjusted odds of extreme PTB (adjusted OR, 1.18 [95% CI, 1.07-1.29]).

When assessing the association of each MVI theme with each PTB gestational age category, the higher MVI quintiles had a higher proportion of PTB across all gestational age categories (eTable 3 in Supplement 1). Compared with very low MVI, very high MVI in each of the 6 themes was associated with all of the PTB gestational age categories in unadjusted analyses (eTable 4 in Supplement 1). In adjusted models, very high physical health MVI was associated with greater odds of extreme, moderate, and late PTB, and very high socioeconomic MVI was associated with extreme PTB (adjusted OR, 1.13 [95% CI, 1.05-1.22]). For the mental health and substance abuse and general health care themes, however, very high mental health and substance abuse MVI was associated with late PTB (adjusted OR, 1.10 [95% CI, 1.03-1.17]) and moderate PTB (adjusted OR, 1.12 [95% CI, 1.03-1.23]), and very high general health care MVI was associated with late PTB (adjusted OR, 1.10 [95% CI, 1.03-1.18]) (eTable 4 in Supplement 1). Associations of quintiles of each MVI theme with PTB gestational age categories are reported in eTables 5 to 10 in Supplement 1. In the sensitivity analysis excluding race and ethnicity from the model, results were similar though with less attenuation of the increased odds for PTB as MVI quintile increased (eFigure in Supplement 1).

## Discussion

We found that the MVI, a new measure of maternal community factors, was associated with PTB in the US. For extreme preterm birth, each increasing quintile of MVI was associated with a greater point estimate. Furthermore, the MVI themes of physical health, mental health and substance abuse, and general health care were consistently associated with PTB; notably, physical health was associated with extreme PTB, and mental health and substance abuse and general health care were associated with late PTB. Collectively, these findings demonstrate concurrent validity and support the MVI as a novel, useful tool to measure the association of a myriad of county-level factors with PTB.

The MVI was associated with PTB, both overall and extreme PTB, adjusted for individual maternal characteristics. The larger point estimates of the MVI with PTB at higher MVI quintiles suggest a potential dose-response association, supporting face validity of the MVI and its association with PTB. Additionally, the point estimate for MVI was largest with respect to extreme PTB, consistent with prior literature of other community-level risk factors.^23^ The MVI may be useful for PTB studies with limited individual-level variables. While PTB has implications for birth parent and infant health, further study of the association of MVI with other birth parent and child health outcomes is needed.

Although the SVI has been used to study community factors associated with PTB,^23^ maternal and perinatal risk factors are unique. The SVI measures community-level risk factors in disaster management and captures 4 themes: (1) socioeconomic determinants; (2) household composition and disability; (3) minority status and language; and (4) housing type and transportation.^22^ The MVI adds relevant indicators such as food insecurity and air pollution as well as novel themes, including physical health, mental health and substance abuse, reproductive health care, and general health care. The MVI may be an ideal index to measure unique facets of county-level community risk factors for PTB. Increasing awareness of the different domains of the SVI vs the MVI may help researchers select the most relevant index for future studies.

Association patterns of the MVI themes of physical health, mental health and substance abuse, and general health care with PTB highlight potential community-specific opportunities to intervene for PTB reduction. In this study, high MVI in area-level indicators of physical health remained associated with PTB in adjusted analyses. This theme is composed of indicators that are known PTB risk factors, including county-level rates of hypertension, diabetes, obesity, and sexually transmitted diseases, which are all major public health concerns.^35,37,45,46^ Funding community interventions to optimize prepregnancy care via state expansion of Medicaid may decrease community vulnerability in this domain.^47,48,49^ High general health care MVI, comprised of quality and accessibility indicators, also remained associated with PTB in adjusted analyses. Improving access to high-quality perinatal care in counties with high MVI in this theme, such as rural areas, may reduce PTB by providing risk-appropriate care to prevent PTB and associated morbidity.^50,51^ Finally, high MVI in the mental health and substance abuse theme remained associated with PTB in adjusted analyses, consistent with prior literature.^52^ Access to substance abuse treatment may reduce associated PTB.^53^ Analyses of the individual MVI themes and gestational age categories suggest that targeting interventions to different MVI themes may differentially affect PTB.

Compared with very low MVI, we did not detect associations of high or low MVI with PTB in adjusted analyses, which may influence the perceived validity of the MVI. However, our thorough adjustment for variables including race and ethnicity and insurance may be overadjustments; one reason for racial disparities in PTB may be due to segregation and differential access to community resources that may be captured within the MVI. The sensitivity analysis without race and ethnicity in the model indicate the need for a separate, critical examination of the potentially differential effect of MVI on PTB risk by race and ethnicity and whether MVI functions as a measure of structural discrimination. Nevertheless, high MVI was consistently associated with increasing severity of PTB. While the themes of physical environment, reproductive health care, and socioeconomic determinants were not associated with PTB in adjusted analyses, further investigation into the association between these themes and other perinatal and infant health outcomes is warranted. Strong evidence links characteristics of the physical environment, specifically measures of pollution, to PTB.^54^ Environmental disparities, including harmful pollution exposure, are due to structural racism.^55^ This is evident in this national cohort, because with each increasing MVI quintile there were higher proportions of non-Hispanic Black birthing people and markedly lower proportions of non-Hispanic White birthing people, which supports the need for antiracism in community programming and policies. Targets of reproductive health care, such as interpregnancy interval, similarly have strong associations with PTB.^56^ County-level variation in socioeconomic determinants in large rural counties or urban counties with diverse microenvironments may have contributed to the association of very high MVI in socioeconomic determinants with extreme PTB only. There is widespread heterogeneity in both the physical environment and public policies across states, such as reproductive policy. Further studies examining variation, by state and within county (eg, at the zip code level), in the MVI by theme may identify appropriate area-specific public health interventions to target PTB.

### Limitations

This study has some limitations. The MVI is a new measure and, to our knowledge, the creators of the MVI have not yet published a report detailing the development process. Many of the indicators that comprise the MVI (derived from data collected between 2016 and 2021) are concurrent with the outcome data (PTB in 2018), yet not all MVI indicators precede the outcome, which may introduce bias such as time-varying confounding. While it seems unlikely that community-level rates of hypertension or poverty would change drastically year to year, future studies to quantify changes in MVI indicators over time would be informative. We completed several analyses, and stratified analyses may have lacked power to confirm associations by MVI theme and PTB category. Additionally, we did not adjust for multiple comparisons in our secondary, exploratory analyses of MVI themes and PTB categories. While some associations were significant, we cannot rule out other associations that may emerge as significant over time with examination of additional years of data. While our models include some patient-level characteristics, unmeasured confounding is likely present, and the addition of other variables could change the study findings. For example, disorders of placentation that may clinically necessitate PTB were not accounted for in this study. Notably, the MVI is only available at the county level, which does not capture variability within counties. However, the MVI may be useful to identify appropriate county-level public health interventions.

Despite these limitations, this study uses a national data set of birth certificate data to demonstrate the association of the MVI with overall PTB and extreme PTB specifically. Relevant themes unique to the MVI, including physical health, mental health and substance abuse, and general health care, were associated with PTB. These findings provide preliminary data to support the MVI as a useful, novel county-level measure for maternal community-level risk factors for PTB. Further research examining the geographic variation of MVI and PTB is warranted to better understand community, county, and state-specific PTB risk factors.

## Conclusions

The MVI, a county-level index measuring maternal community risk factors for adverse maternal outcomes, is associated with the perinatal outcome of PTB. The association of MVI with the risk of PTB was highest for extreme PTB, and the MVI themes of physical health, mental health and substance abuse, and general health care may be most important for PTB. Our findings support the MVI as a useful index to examine the role of maternal community-level risk factors in PTB. Variability in this measure highlights the importance of unique state and county-specific policies to address community-specific maternal risks of PTB.

## Notes

Supplement 1. **eTable 1.** Maternal Vulnerability Index Themes, Subthemes, and Associated Indicators **eTable 2.** Area and Perinatal Characteristics of the Included 3 659 099 Infants by Gestational Age Category **eTable 3.** Distribution of Preterm Birth Rates by Gestational Age Categories for Each Quintile of MVI Themes **eTable 4.** Unadjusted and Adjusted Associations of Very High MVI Compared With Very Low MVI for Each MVI Theme and Gestational Age Category Compared With Term Births **eTable 5.** Models of Association Between Reproductive Health Care MVI Theme and PTB Gestational Age Category **eTable 6.** Models of Association Between Physical Health MVI Theme and PTB Gestational Age Category **eTable 7.** Models of Association Between Mental Health and Substance Abuse MVI Theme and PTB Gestational Age Category **eTable 8.** Models of Association Between General Health Care MVI Theme and PTB Gestational Age Category **eTable 9.** Models of Association Between Socioeconomic MVI Theme and PTB Gestational Age Category **eTable 10.** Models of Association Between Physical Environment MVI Theme and PTB Gestational Age Category **eFigure.** Unadjusted and Adjusted Associations of MVI With Preterm Birth by Gestational Age Categories Compared With Term Births

Supplement 2.
Data Sharing Statement
