# Supplementary material for: County-Level Maternal Vulnerability and Preterm Birth in the US
Source: JAMA Netw Open. 2023 May 25;6(5):e2315306. doi: 10.1001/jamanetworkopen.2023.15306 (PMC10214038; doi:10.1001/jamanetworkopen.2023.15306)
Supplement: Supplement 1. — eTable 1. Maternal Vulnerability Index Themes, Subthemes, and Associated Indicators eTable 2. Area and Perinatal Characteristics of the Included 3 659 099 Infants by Gestational Age Category eTable 3. Distribution of Preterm Birth Rates by Gestational Age Categories for Each Quintile of MVI Themes eTable 4. Unadjusted and Adjusted Associations of Very High MVI Compared With Very Low MVI for Each MVI Theme and Gestational Age Category Compared With Term Births eTable 5. Models of Association Between Reproductive Health Care MVI Theme and PTB Gestational Age Category eTable 6. Models of Association Between Physical Health MVI Theme and PTB Gestational Age Category eTable 7. Models of Association Between Mental Health and Substance Abuse MVI Theme and PTB Gestational Age Category eTable 8. Models of Association Between General Health Care MVI Theme and PTB Gestational Age Category eTable 9. Models of Association Between Socioeconomic MVI Theme and PTB Gestational Age Category eTable 10. Models of Association Between Physical Environment MVI Theme and PTB Gestational Age Category eFigure. Unadjusted and Adjusted Associations of MVI With Preterm Birth by Gestational Age Categories Compared With Term Births [file jamanetwopen-e2315306-s001.pdf]

Supplementary Online Content

Salazar EG, Montoya-Williams D, Passarella M, et al. County-level maternal vulnerability and preterm birth in the US. *JAMA Netw Open*. 2023;6(5):e2315306. doi:10.1001/jamanetworkopen.2023.15306

eTable 1. Maternal Vulnerability Index Themes, Subthemes, and Associated Indicators
eTable 2. Area and Perinatal Characteristics of the Included 3 659 099 Infants by Gestational Age Category
eTable 3. Distribution of Preterm Birth Rates by Gestational Age Categories for Each Quintile of MVI Themes
eTable 4. Unadjusted and Adjusted Associations of Very High MVI Compared With Very Low MVI for Each MVI Theme and Gestational Age Category Compared With Term Births
eTable 5. Models of Association Between Reproductive Health Care MVI Theme and PTB Gestational Age Category
eTable 6. Models of Association Between Physical Health MVI Theme and PTB Gestational Age Category
eTable 7. Models of Association Between Mental Health and Substance Abuse MVI Theme and PTB Gestational Age Category
eTable 8. Models of Association Between General Health Care MVI Theme and PTB Gestational Age Category
eTable 9. Models of Association Between Socioeconomic MVI Theme and PTB Gestational Age Category
eTable 10. Models of Association Between Physical Environment MVI Theme and PTB Gestational Age Category
eFigure. Unadjusted and Adjusted Associations of MVI With Preterm Birth by Gestational Age Categories Compared With Term Births

This supplementary material has been provided by the authors to give readers additional information about their work.

© 2023 Salazar EG et al. *JAMA Network Open*.

eTable 1. Maternal Vulnerability Index Themes, Subthemes, and Associated Indicators

Theme	Subtheme	Indicator
Reproductive healthcare	Family planning needs	Female contraceptive clients served at publicly funded clinics per 100,000 women of reproductive age (13-44 years)
	Access to abortions	Minimum distance to nearest abortion clinic for women of reproductive age
		Count of supportive abortion policies
	Public reproductive health funding	Public expenditures for family planning client services per capita
	Reproductive health system capacity	Ratio of OBGYN providers per women of reproductive age
		Ratio of nurse midwives per women of reproductive age
		Ratio of newborn bassinets per women of reproductive age
Physical health	Hypertension	Percent of adult population with high blood pressure
	Diabetes	Percent of female adult population with diabetes (>20 years by county and >18 years by state)
	Obesity	Percent of female adult population that is obese (age >20)
	Prevalence of STIs	Prevalence of gonorrhea
		Prevalence of primary or secondary syphilis
		Prevalence of chlamydia
		Prevalence of hepatitis B
	HIV Prevalence	Prevalence of HIV in the female population (≥13 years)
	Self-Rated Health	Percent of the adult population reporting poor or fair health
Mental health and substance abuse	Stress	Percent of adult population reporting frequent mental distress
		Percent of female worker population with a long commute (>30 minutes)
	Mental health status	Percent of adult population reporting any mental illness
		Percent of adult population ever diagnosed with depression
	Accessibility	Mental health providers per capita
	Substance abuse	Percent of adults that smoke
		Age adjusted overdose death rate
General healthcare	Affordability	Postpartum extension status
		Income eligibility limit for pregnant individuals
		Medicaid expansion status
		Percent of women of reproductive age who are uninsured (19-44 years)
	Accessibility	Distance to the nearest hospital
	Care seeking behavior	Percent of the adult population that reported a routine physical checkup

© 2023 Salazar EG et al. *JAMA Network Open*.

		Percent of the adult population that reported having a primary care physician
	Quality	Prevention Quality Indicator
Socioeconomic determinants	Educational attainment	Percent of women of reproductive age (≥25 years) with a bachelor's degree
		Percent of women of reproductive age (18-44 years) with no high school degree
	Minority status	Percent of the population that speaks English less than well
	Poverty	Percent of women of reproductive age (15-44 years) that live under poverty
	Food insecurity	Percent of the general population suffering from food insecurity
	Social Capital	Percent of households that are single female headed
		Social capital index
Physical environment	Housing	Percent of households with severe housing problems
	Violence	Violent crime rate per 100,000 population
	Transportation	Percent of adult female population (≥16 years) with no access to a vehicle
		Transit Connectivity Index (TCI)
	Air pollution	Concentration of particulate matter of size ≤2.5 micrometers

Adapted from Surgo Ventures (2021). The US Maternal Vulnerability Index (MVI) Methodology, Version 3.

**eTable 2.** Area and Perinatal Characteristics of the Included 3 659 099 Infants by Gestational Age Category					
Gestational Age Category	Extreme PTB	Very PTB	Moderate PTB	Late PTB	Term
Births	N=16,719 (%)	N=25,728 (%)	N=32,241 (%)	N=224,159 (%)	N=3,360,252 (%)
MVI[a]					
Very Low	3,376 (20.2)	5,707 (22.2)	7,183 (22.3)	52,674 (23.5)	911,380 (27.1)
Low	3,681 (22.0)	5,756 (22.4)	7,080 (22.0)	49,805 (22.2)	797,147 (23.7)
Moderate	3,524 (21.1)	5,276 (20.5)	6,742 (20.9)	45,357 (20.2)	671,241 (20.0)
High	4,321 (25.8)	6,243 (24.3)	7,852 (24.4)	52,841 (23.6)	703,728 (20.9)
Very High	1,817 (10.9)	2,746 (10.7)	3,384 (10.5)	23,482 (10.5)	276,756 (8.2)
Urban Influence Code[a]					
Metropolitan	14,571 (87.2)	22,118 (86.0)	27,784 (86.2)	191,737 (85.5)	2,906,868 (86.5)
Micropolitan	1,273 (7.6)	2,153 (8.4)	2,667 (8.3)	19,285 (8.6)	272,419 (8.1)
Noncore	875 (5.2)	1,457 (5.7)	1,790 (5.6)	13,137 (5.9)	180,965 (5.4)
Census Regions[a]					
Northeast	2,398 (14.3)	3,894 (15.1)	4,847 (15.0)	31,927 (14.2)	541,885 (16.1)
Midwest	3,346 (20.0)	5,318 (20.7)	6,598 (20.5)	47,406 (21.2)	706,339 (21.0)
South	7,811 (46.7)	11,318 (44.0)	14,312 (44.4)	96,299 (43.0)	1,305,614 (38.9)
West	3,164 (18.9)	5,198 (20.2)	6,484 (20.1)	48,527 (21.7)	806,414 (24.0)
Birth weight, g (mean, SD)[a]	785 (292)	1457 (462)	1991 (441)	2661 (495)	3377 (460)
Gestational age, weeks (mean, SD)[a]	25 (2)	30 (1)	33 (0.5)	35 (1)	39 (1)
Maternal Race/Ethnicity[a]					
American Native/American Indian	123 (0.7)	247 (1.0)	356 (1.1)	2,178 (1.0)	25,702 (0.8)
Asian/Pacific Islander	859 (5.1)	1,510 (5.9)	1,845 (5.7)	13,770 (6.1)	230,957 (6.9)
Hispanic	3,878 (23.2)	5,835 (22.7)	7,476 (23.2)	54,564 (24.3)	791,147 (23.5)
Multiracial	425 (2.5)	605 (2.4)	741 (2.3)	5,294 (2.4)	74,098 (2.2)
Non-Hispanic Black	5,747 (34.4)	6,951 (27.0)	7,541 (23.4)	42,166 (18.8)	469,395 (14.0)
Non-Hispanic White	5,687 (34.0)	10,580 (41.1)	14,282 (44.3)	106,187 (47.4)	1,768,953 (52.6)
Maternal Age (years)[a]					
<20	1,189 (7.1)	1,530 (6.0)	1,823 (5.7)	11,972 (5.3)	161,599 (4.8)
20-24	3,519 (21.1)	5,112 (19.9)	6,149 (19.1)	43,310 (19.3)	649,795 (19.3)
25-34	8,653 (51.8)	13,538 (52.6)	17,099 (53.0)	121,573 (54.2)	1,951,328 (58.1)
≥35	3,358 (20.1)	5,548 (21.6)	7,170 (22.2)	47,304 (21.1)	597,530 (17.8)
Maternal Insurance[a]					
Private	6,429 (38.5)	10,321 (40.1)	13,128 (40.7)	97,531 (43.5)	1,671,984 (49.8)
Medicaid	8,714 (52.1)	13,175 (51.2)	16,274 (50.5)	108,221 (48.3)	1,398,830 (41.6)
Self-pay	793 (4.7)	1,021 (4.0)	1,299 (4.0)	8,392 (3.7)	141,261 (4.2)
Other	628 (3.8)	1,004 (3.9)	1,305 (4.1)	8,601 (3.8)	129,090 (3.8)
Missing	155 (0.9)	207 (0.8)	235 (0.7)	1,414 (0.6)	19,087 (0.6)
Maternal Education[a]					
8th grade or less	439 (2.6)	764 (3.0)	1,052 (3.3)	7,327 (3.3)	105,314 (3.1)
9-12th grade, no degree	2,141 (12.8)	3,307 (12.9)	4,094 (12.7)	26,665 (11.9)	311,630 (9.3)
High School/GED	5,325 (31.9)	7,709 (30.0)	9,449 (29.3)	63,583 (28.4)	850,772 (25.3)
Some college	5,018 (30.0)	7,623 (29.6)	9,439 (29.3)	65,861 (29.4)	939,772 (28.0)
Bachelor's degree or higher	3,332 (19.9)	5,830 (22.7)	7,706 (22.9)	57,498 (25.7)	1,111,652 (33.1)
Missing	464 (2.8)	495 (1.9)	501 (1.6)	3,225 (1.4)	41,112 (1.2)
Kotelchuk Index[a]					
Inadequate	2,708 (16.2)	3,476 (13.5)	3,997 (12.4)	23,939 (10.7)	271,215 (8.1)
Intermediate	2,001 (12.0)	3,249 (12.6)	5,799 (18.0)	32,212 (14.4)	862,420 (25.7)
Adequate	4,477 (26.8)	5,904 (23.0)	6,877 (21.3)	63,042 (28.1)	1,628,126 (48.5)
Adequate+	6,130 (36.7)	11,445 (44.5)	13,874 (43.0)	96,606 (43.1)	521,911 (15.5)
Missing	1,403 (8.4)	1,654 (6.4)	1,694 (5.3)	8,360 (3.7)	76,580 (2.3)
Nulliparous[a]	8,085 (48.4)	11,047 (42.9)	12,979 (40.3)	83,022 (37.0)	1,287,614 (38.3)
BMI (kg/m^2)[a]					
Underweight (<18.5)	547 (3.3)	948 (3.7)	1,270 (3.9)	8,435 (3.8)	103,606 (3.1)
Normal (18.5-24.9)	5,037 (30.1)	8,755 (34.0)	11,473 (35.6)	82,770 (36.9)	1,404,620 (41.8)

© 2023 Salazar EG et al. *JAMA Network Open*.

Overweight (25.0-29.9)	3,992 (23.9)	6,175 (24.0)	7,842 (24.3)	55,181 (24.6)	878,558 (26.2)
Obesity (≥30)	6,032 (36.1)	8,541 (33.2)	10,340 (32.1)	70,792 (31.6)	903,101 (26.9)
Missing	1,111 (6.7)	1,309 (5.1)	1,316 (4.1)	6,981 (3.1)	70,367 (2.1)
Any smoking during pregnancy[a]	1,515 (9.1)	2,631 (10.2)	3,281 (10.2)	21,259 (9.5)	208,089 (6.2)
Pre-existing hypertension[a]	1,132 (6.8)	2,005 (7.8)	2,187 (6.8)	10,810 (4.8)	58,047 (1.7)
Gestational hypertension	1,938 (11.6)	5,218 (20.3)	6,485 (20.1)	35,733 (15.9)	204,728 (6.1)
Gestational diabetes	623 (3.7)	1,910 (7.4)	2,941 (9.1)	21,823 (9.7)	213,510 (6.4)

[a] $p<0.001$

GA: Gestational Age; PTB: Preterm Birth; AN: American Native; AI: American Indian

The Kotelchuck index, or Adequacy of Prenatal Care Utilization Index, describes the adequacy of received prenatal care using birth certificate data. Inadequate indicates <50% of expected visits, intermediate indicates 50-79% of expected visits, adequate indicates 80-109% of expected visits, and adequate+ indicates 110% or more expected visits.

© 2023 Salazar EG et al. *JAMA Network Open*.

**eTable 3.** Distribution of Preterm Birth Rates by Gestational Age Categories for Each Quintile of MVI Themes					
	**Very Low MVI**	**Low MVI**	**Moderate MVI**	**High MVI**	**Very High MVI**
**MVI Reproductive Healthcare**[a]	**N=1,500,952**	**N=829,786**	**N=604,297**	**N=547,188**	**N=176,876**
Preterm birth	113,575 (7.6)	69,227 (8.3)	53,274 (8.8)	47,124 (8.6)	15,647 (8.9)
Extremely preterm	6,450 (0.4)	3,916 (0.5)	3,169 (0.5)	2,407 (0.4)	777 (0.4)
Very preterm	10,054 (0.7)	6,062 (0.7)	4,523 (0.8)	3,827 (0.7)	1,262 (0.7)
Moderate preterm	12,390 (0.8)	7,559 (0.9)	5,758 (1.0)	4,935 (0.9)	1,599 (0.9)
Late preterm	84,681 (5.6)	51,690 (6.2)	39,824 (6.6)	35,955 (6.6)	12,009 (6.8)
Term	1,387,377 (92.4)	760,559 (91.7)	551,023 (91.2)	500,064 (91.4)	161,229 (91.2)
**MVI Physical Health**[a]	**N=807,501**	**N=799,035**	**N=1,025,285**	**N=594,606**	**N=432,672**
Preterm birth	56,179 (7.0)	60,148 (7.5)	85,683 (8.4)	52,837 (8.9)	44,000 (10.2)
Extremely preterm	2,592 (0.3)	3,118 (0.4)	5,019 (0.5)	3,061 (0.5)	2,929 (0.7)
Very preterm	4,546 (0.6)	5,102 (0.6)	7,322 (0.7)	4,704 (0.8)	4,054 (0.9)
Moderate preterm	5,632 (0.7)	6,385 (0.8)	9,523 (0.9)	5,795 (1.0)	4,906 (1.1)
Late preterm	43,409 (5.4)	45,543 (5.7)	63,819 (6.2)	39,277 (6.6)	32,111 (7.4)
Term	751,322 (93.0)	738,887 (92.5)	939,602 (91.6)	541,769 (91.1)	388,672 (89.8)
**MVI Mental Health/Substance Abuse**[a]	**N=1,061,489**	**N=898,961**	**N=745,456**	**N=614,351**	**N=338,842**
Preterm birth	77,274 (7.3)	72,574 (8.1)	62,436 (8.4)	54,644 (8.9)	31,919 (9.4)
Extremely preterm	4,091 (0.4)	3,931 (0.4)	3,710 (0.5)	3,263 (0.5)	1,724 (0.5)
Very preterm	6,549 (0.6)	6,027 (0.7)	5,467 (0.7)	4,912 (0.8)	2,773 (0.8)
Moderate preterm	7,992 (0.8)	7,912 (0.9)	6,889 (0.9)	5,980 (1.0)	3,468 (1.0)
Late preterm	58,642 (5.5)	54,704 (6.1)	46,370 (6.2)	40,489 (6.6)	23,954 (7.1)
Term	984,215 (92.7)	826,387 (91.9)	683,020 (91.6)	559,707 (91.1)	306,923 (90.6)
**MVI General Healthcare**[a]	**N=1,566,005**	**N=787,618**	**N=539,455**	**N=518,550**	**N=247,471**
Preterm birth	120,257 (7.7)	63,543 (8.1)	45,812 (8.5)	45,480 (8.8)	23,755 (9.6)
Extremely preterm	6,673 (0.4)	3,601 (0.5)	2,490 (0.5)	2,694 (0.5)	1,261 (0.5)
Very preterm	10,538 (0.7)	5,433 (0.7)	3,927 (0.7)	3,927 (0.8)	1,903 (0.8)
Moderate preterm	13,113 (0.8)	6,692 (0.9)	5,025 (0.9)	4,961 (1.0)	2,450 (1.0)
Late preterm	89,933 (5.7)	47,817 (6.1)	34,370 (6.4)	33,898 (6.5)	18,141 (7.3)
Term	1,445,748 (92.3)	724,075 (91.9)	493,643 (91.5)	473,070 (91.2)	223,716 (90.4)
**MVI Socioeconomic Determinants**[a]	**N=477,213**	**N=811,529**	**N=775,222**	**N=1,035,632**	**N=559,503**
Preterm birth	33,599 (7.0)	60,699 (7.5)	63,149 (8.2)	88,523 (8.6)	52,877 (9.5)
Extremely preterm	1,518 (0.3)	3,156 (0.4)	3,603 (0.5)	5,201 (0.5)	3,241 (0.6)
Very preterm	2,631 (0.6)	5,106 (0.6)	5,547 (0.7)	7,820 (0.8)	4,624 (0.8)
Moderate preterm	3,509 (0.7)	6,314 (0.8)	6,910 (0.9)	9,624 (0.9)	5,884 (1.1)
Late preterm	25,941 (5.4)	46,123 (5.7)	47,089 (6.1)	65,878 (6.4)	39,128 (7.0)
Term	443,614 (93.0)	750,830 (92.5)	712,073 (91.9)	947,109 (91.5)	506,626 (90.6)
**MVI Physical Environment**[a]	**N=217,985**	**N=468,729**	**N=790,079**	**N=925,874**	**N=1,256,432**
Preterm birth	15,841 (7.3)	34,450 (7.4)	62,037 (7.9)	77,123 (8.3)	109,396 (8.7)
Extremely preterm	735 (0.3)	1,623 (0.4)	3,343 (0.4)	4,281 (0.5)	6,737 (0.5)
Very preterm	1,218 (0.6)	2,808 (0.6)	5,211 (0.7)	6,680 (0.7)	9,811 (0.8)
Moderate preterm	1,569 (0.7)	3,546 (0.8)	6,679 (0.9)	8,276 (0.9)	12,171 (1.0)
Late preterm	12,319 (5.7)	26,473 (5.7)	46,804 (5.9)	57,886 (6.3)	80,677 (6.4)
Term	202,144 (92.7)	434,279 (92.7)	728,042 (92.2)	848,751 (91.7)	1,147,036 (91.3)

[a]p<0.001 when comparing preterm birth (<37 weeks) across MVI quintiles and between gestational age categories (extreme, very, moderate, late, and term) across MVI quintiles.
Gestational age categories: Preterm birth (<37 weeks), extremely preterm (<28 weeks), very preterm (28-<32 weeks), moderate preterm (32-<34 weeks), late preterm (34-<37 weeks), term (≥37 weeks)
Abbreviations: MVI: Maternal Vulnerability Index, wks: weeks

© 2023 Salazar EG et al. *JAMA Network Open*.

eTable 4. Unadjusted and Adjusted Associations of Very High MVI Compared With Very Low MVI for Each MVI Theme and Gestational Age Category Compared With Term Births

	**Extreme PTB**		**Very PTB**		**Moderate PTB**		**Late PTB**	
**MVI Theme**	OR (95% CI)	aOR (95% CI)	OR (95% CI)	aOR (95% CI)	OR (95% CI)	aOR (95% CI)	OR (95% CI)	aOR (95% CI)
Reproductive Healthcare	1.04 (0.94,1.14)	1.06 (0.95,1.18)	1.08* (1.00,1.17)	0.95 (0.86,1.05)	1.11[b] (1.03,1.20)	1.00 (0.91,1.09)	1.22* (1.17,1.27)	1.04 (0.98,1.11)
Physical Health	2.18[a] (2.03,2.35)	1.26[a] (1.16,1.37)	1.72[a] (1.64,1.82)	1.06 (0.99,1.14)	1.68[a] (1.61,1.77)	1.12[b] (1.04,1.21)	1.43[a] (1.38,1.48)	1.07[b] (1.02,1.13)
Mental Health / Substance Abuse	1.35[a] (1.22,1.49)	1.06 (0.95,1.19)	1.36[a] (1.28,1.44)	1.04 (0.94,1.15)	1.39[a] (1.32,1.47)	1.12[b] (1.03,1.23)	1.31[a] (1.27,1.35)	1.10[b] (1.03,1.17)
General Healthcare	1.22[a] (1.12,1.33)	1.08 (0.97,1.21)	1.17[a] (1.10,1.24)	0.99 (0.90,1.10)	1.21[a] (1.13,1.29)	1.01 (0.93,1.10)	1.30[a] (1.23,1.38)	1.10[b] (1.03,1.18)
Socioeconomic Determinants	1.87[a] (1.74,2.01)	1.13[b] (1.05,1.22)	1.54[a] (1.44,1.64)	1.03 (0.96,1.10)	1.47[a] (1.39,1.55)	1.03 (0.96,1.10)	1.32[a] (1.26,1.38)	0.99 (0.95,1.04)
Physical Environment	1.62[a] (1.44,1.81)	1.02 (0.92,1.13)	1.42[a] (1.31,1.53)	0.99 (0.92,1.08)	1.37[a] (1.26,1.49)	0.99 (0.91,1.07)	1.15[a] (1.10,1.21)	0.96 (0.92,1.00)

[a]p<0.001, [b]p<0.05
MVI: Maternal Vulnerability Index, PTB: Preterm Birth, RRR: Relative risk ratio, aRRR: Adjusted relative risk ratio. Adjusted for age, race/ethnicity, insurance, education, Kotelchuk index of adequate prenatal care, body mass index, nulliparity, pre-existing hypertension, smoking during pregnancy, rurality, infant sex, and census region. Term birth serves as a reference.
The output from the Stata mlogit model is expressed as a ratio of relative risk which but for simplicity is reported as an odds ratio.

© 2023 Salazar EG et al. *JAMA Network Open*.

**eTable 5.** Models of Association Between Reproductive Health Care MVI Theme and PTB Gestational Age Category		
**Outcomes**	**Unadjusted OR (95% CI)**	**Adjusted OR (95% CI)**
**Extreme Preterm Birth**		
Very Low MVI	Reference	Reference
Low MVI	1.11 (1.02,1.21)[b]	1.02 (0.96,1.10)
Moderate MVI	1.24 (1.14,1.34)[a]	1.07 (0.99,1.15)
High MVI	1.04 (0.95,1.12)	0.98 (0.89,1.07)
Very High MVI	1.04 (0.94,1.14)	1.06 (0.95,1.18)
**Very Preterm Birth**		
Very Low MVI	Reference	Reference
Low MVI	1.10 (1.03,1.17)[b]	1.00 (0.95,1.05)
Moderate MVI	1.13 (1.07,1.19)[a]	0.95 (0.88,1.01)
High MVI	1.06 (0.99,1.12)	0.94 (0.87,1.01)
Very High MVI	1.08 (1.00,1.17)[b]	0.95 (0.86,1.05)
**Moderate Preterm Birth**		
Very Low MVI	Reference	Reference
Low MVI	1.11 (1.05,1.18)[a]	1.02 (0.97,1.08)
Moderate MVI	1.17 (1.09,1.25)[a]	0.99 (0.93,1.06)
High MVI	1.11 (1.03,1.19)[b]	0.99 (0.92,1.07)
Very High MVI	1.11 (1.03,1.20)[b]	1.00 (0.91,1.09)
**Late Preterm Birth**		
Very Low MVI	Reference	Reference
Low MVI	1.11 (1.08,1.15)[a]	1.03 (0.99,1.06)
Moderate MVI	1.18 (1.14,1.23)[a]	1.02 (0.97,1.07)
High MVI	1.18 (1.12,1.24)[a]	1.03 (0.98,1.09)
Very High MVI	1.22 (1.17,1.27)[a]	1.04 (0.98,1.11)
**Term Birth**		
	Reference	Reference

[a]$p \leq 0.001$, [b]$p \leq 0.05$

GA: Gestational Age; PTB: Preterm Birth

MVI is the Maternal Vulnerability Index. Very Low MVI is 0-20. Low MVI is 20-40. Moderate MVI is 40-60. High MVI is 60-80. Very High MVI is 80-100. Adjusted for sex, race, age, insurance, education, adequacy of prenatal care, nulliparous status, pre-existing hypertension, body mass index, smoking, and state.

The output from the Stata mlogit model is expressed as a ratio of relative risk which but for simplicity is reported as an odds ratio.

© 2023 Salazar EG et al. *JAMA Network Open*.

**eTable 6.** Models of Association Between Physical Health MVI Theme and PTB Gestational Age Category		
**Outcomes**	**Unadjusted OR (95% CI)**	**Adjusted OR (95% CI)**
**Extreme Preterm Birth**		
Very Low MVI	Reference	Reference
Low MVI	1.22 (1.14,1.31)[a]	1.07 (1.01,1.13)[b]
Moderate MVI	1.55 (1.44,1.66)[a]	1.13 (1.05,1.21)[a]
High MVI	1.64 (1.51,1.77)[a]	1.16 (1.08,1.25)[a]
Very High MVI	2.18 (2.03,2.35)[a]	1.26 (1.16,1.37)[a]
**Very Preterm Birth**		
Very Low MVI	Reference	Reference
Low MVI	1.14 (1.09,1.19)[a]	1.01 (0.96,1.06)
Moderate MVI	1.29 (1.23,1.35)[a]	1.00 (0.95,1.06)
High MVI	1.43 (1.36,1.51)[a]	1.06 (1.00,1.14)
Very High MVI	1.72 (1.64,1.82)[a]	1.06 (0.99,1.14)
**Moderate Preterm Birth**		
Very Low MVI	Reference	Reference
Low MVI	1.15 (1.10,1.21)[a]	1.03 (0.97,1.10)
Moderate MVI	1.35 (1.28,1.43)[a]	1.08 (1.02,1.14)[b]
High MVI	1.43 (1.36,1.50)[b]	1.10 (1.03,1.17)[b]
Very High MVI	1.68 (1.61,1.77)[b]	1.12 (1.04,1.21)[b]
**Late Preterm Birth**		
Very Low MVI	Reference	Reference
Low MVI	1.07 (1.03,1.10)[a]	1.00 (0.95,1.04)
Moderate MVI	1.18 (1.13,1.22)[a]	1.01 (0.97,1.06)
High MVI	1.25 (1.21,1.31)[a]	1.05 (1.00,1.11)
Very High MVI	1.43 (1.38,1.48)[a]	1.07 (1.02,1.13)[b]
**Term Birth**		
	Reference	Reference

[a]p<0.001, [b]p<0.05

GA: Gestational Age; PTB: Preterm Birth

MVI is the Maternal Vulnerability Index. Very Low MVI is 0-20. Low MVI is 20-40. Moderate MVI is 40-60. High MVI is 60-80. Very High MVI is 80-100. Adjusted for sex, race, age, insurance, education, adequacy of prenatal care, nulliparous status, pre-existing hypertension, body mass index, smoking, and state.

The output from the Stata mlogit model is expressed as a ratio of relative risk which but for simplicity is reported as an odds ratio.

© 2023 Salazar EG et al. *JAMA Network Open*.

eTable 7. Models of Association Between Mental Health and Substance Abuse MVI Theme and PTB Gestational Age Category

**Outcomes**	**Unadjusted OR (95% CI)**	**Adjusted OR (95% CI)**
**Extreme Preterm Birth**		
Very Low MVI	Reference	Reference
Low MVI	1.14 (1.04,1.26)[b]	1.00 (0.93,1.07)
Moderate MVI	1.31 (1.20,1.42)[a]	1.02 (0.95,1.10)
High MVI	1.40 (1.29,1.52)[a]	1.08 (0.99,1.18)
Very High MVI	1.35 (1.22,1.49)[a]	1.06 (0.95,1.19)
**Very Preterm Birth**		
Very Low MVI	Reference	Reference
Low MVI	1.10 (1.03,1.16)[b]	1.01 (0.96,1.07)
Moderate MVI	1.20 (1.14,1.27)[a]	1.01 (0.94,1.07)
High MVI	1.32 (1.25,1.40)[a]	1.05 (0.97,1.13)
Very High MVI	1.36 (1.28,1.44)[a]	1.04 (0.94,1.15)
**Moderate Preterm Birth**		
Very Low MVI	Reference	Reference
Low MVI	1.18 (1.10,1.26)[a]	1.07 (1.01,1.13)[b]
Moderate MVI	1.24 (1.18,1.31)[a]	1.07 (1.00,1.14)[b]
High MVI	1.32 (1.25,1.38)[a]	1.11 (1.03,1.19)[b]
Very High MVI	1.39 (1.32,1.47)[a]	1.12 (1.03,1.23)[b]
**Late Preterm Birth**		
Very Low MVI	Reference	Reference
Low MVI	1.11 (1.06,1.17)[a]	1.03 (0.99,1.08)
Moderate MVI	1.14 (1.10,1.18)[a]	1.01 (0.97,1.06)
High MVI	1.21 (1.18,1.25)[a]	1.07 (1.02,1.13)[b]
Very High MVI	1.31 (1.27,1.35)[a]	1.10 (1.03,1.17)[b]
**Term Birth**		
	Reference	Reference

[a]p<0.001, [b]p<0.05

GA: Gestational Age; PTB: Preterm Birth

MVI is the Maternal Vulnerability Index. Very Low MVI is 0-20. Low MVI is 20-40. Moderate MVI is 40-60. High MVI is 60-80. Very High MVI is 80-100. Adjusted for sex, race, age, insurance, education, adequacy of prenatal care, nulliparous status, pre-existing hypertension, body mass index, smoking, and state.

The output from the Stata mlogit model is expressed as a ratio of relative risk which but for simplicity is reported as an odds ratio.

© 2023 Salazar EG et al. *JAMA Network Open*.

**eTable 8.** Models of Association Between General Health Care MVI Theme and PTB Gestational Age Category		
**Outcomes**	**Unadjusted OR (95% CI)**	**Adjusted OR (95% CI)**
**Extreme Preterm Birth**		
Very Low MVI	Reference	Reference
Low MVI	1.08 (0.99,1.17)	1.05 (0.98,1.13)
Moderate MVI	1.09 (0.99,1.20)	1.04 (0.96,1.13)
High MVI	1.23(1.14,1.34)[a]	1.05 (0.95,1.16)
Very High MVI	1.22 (1.12,1.33)[a]	1.08 (0.97,1.21)
**Very Preterm Birth**		
Very Low MVI	Reference	Reference
Low MVI	1.03 (0.97,1.09)	0.99 (0.94,1.05)
Moderate MVI	1.09 (1.02,1.17)[b]	1.03 (0.96,1.11)
High MVI	1.14 (1.07,1.21)[a]	1.01 (0.92,1.10)
Very High MVI	1.17 (1.10,1.24)[a]	0.99 (0.90,1.10)
**Moderate Preterm Birth**		
Very Low MVI	Reference	Reference
Low MVI	1.02 (0.96,1.08)	1.00 (0.96,1.04)
Moderate MVI	1.12 (1.05,1.20)[a]	1.06 (1.00,1.12)[b]
High MVI	1.16 (1.08,1.24)[a]	1.02 (0.94,1.10)
Very High MVI	1.21 (1.13,1.29)[a]	1.01 (0.93,1.10)
**Late Preterm Birth**		
Very Low MVI	Reference	Reference
Low MVI	1.06 (1.03,1.10)[a]	1.01 (0.98,1.05)
Moderate MVI	1.12 (1.08,1.16)[a]	1.04 (1.00,1.08)
High MVI	1.15 (1.10,1.21)[a]	1.04 (0.97,1.11)
Very High MVI	1.30 (1.23,1.38)[a]	1.10 (1.03,1.18)[b]
**Term Birth**		
	Reference	Reference

[a]p<0.001, [b]p<0.05

GA: Gestational Age; PTB: Preterm Birth

MVI is the Maternal Vulnerability Index. Very Low MVI is 0-20. Low MVI is 20-40. Moderate MVI is 40-60. High MVI is 60-80. Very High MVI is 80-100. Adjusted for sex, race, age, insurance, education, adequacy of prenatal care, nulliparous status, pre-existing hypertension, body mass index, smoking, and state.

The output from the Stata mlogit model is expressed as a ratio of relative risk which but for simplicity is reported as an odds ratio.

© 2023 Salazar EG et al. *JAMA Network Open*.

**eTable 9.** Models of Association Between Socioeconomic MVI Theme and PTB Gestational Age Category		
**Outcomes**	**Unadjusted OR (95% CI)**	**Adjusted OR (95% CI)**
**Extreme Preterm Birth**		
Very Low MVI	Reference	Reference
Low MVI	1.23 (1.13,1.34)[a]	1.06 (0.99,1.14)
Moderate MVI	1.48 (1.35,1.61)[a]	1.06 (0.99,1.14)
High MVI	1.60 (1.47,1.75)[a]	1.09 (1.01,1.17)[b]
Very High MVI	1.87 (1.74,2.01)[a]	1.13 (1.05,1.22)[b]
**Very Preterm Birth**		
Very Low MVI	Reference	Reference
Low MVI	1.15 (1.08,1.22)[a]	1.02 (0.96,1.08)
Moderate MVI	1.31 (1.24,1.39)[a]	1.02 (0.96,1.09)
High MVI	1.39 (1.30,1.49)[a]	1.02 (0.95,1.08)
Very High MVI	1.54 (1.44,1.64)[a]	1.03 (0.96,1.10)
**Moderate Preterm Birth**		
Very Low MVI	Reference	Reference
Low MVI	1.06 (1.00,1.13)[b]	0.96 (0.90,1.02)
Moderate MVI	1.23 (1.15,1.30)[a]	0.97 (0.91,1.03)
High MVI	1.28 (1.19,1.38)[a]	0.98 (0.91,1.04)
Very High MVI	1.47 (1.39,1.55)[a]	1.03 (0.96,1.10)
**Late Preterm Birth**		
Very Low MVI	Reference	Reference
Low MVI	1.05 (1.01,1.09)[b]	0.97 (0.93,1.00)
Moderate MVI	1.13 (1.10,1.17)[a]	0.96 (0.93,1.00)
High MVI	1.19 (1.14,1.24)[a]	0.96 (0.92,1.00)[b]
Very High MVI	1.32 (1.26,1.38)[a]	0.99 (0.95,1.03)[b]
**Term Birth**		
	Reference	Reference

[a]p<0.001, [b]p<0.05

GA: Gestational Age; PTB: Preterm Birth

MVI is the Maternal Vulnerability Index. Very Low MVI is 0-20. Low MVI is 20-40. Moderate MVI is 40-60. High MVI is 60-80. Very High MVI is 80-100. Adjusted for sex, race, age, insurance, education, adequacy of prenatal care, nulliparous status, pre-existing hypertension, body mass index, smoking, and state.

The output from the Stata mlogit model is expressed as a ratio of relative risk which but for simplicity is reported as an odds ratio.

© 2023 Salazar EG et al. *JAMA Network Open*.

**eTable 10.** Models of Association Between Physical Environment MVI Theme and PTB Gestational Age Category		
**Outcomes**	**Unadjusted OR (95% CI)**	**Adjusted OR (95% CI)**
**Extreme Preterm Birth**		
Very Low MVI	Reference	Reference
Low MVI	1.03 (0.92,1.15)	0.94 (0.84,1.04)
Moderate MVI	1.26 (1.13,1.41)[a]	0.98 (0.89,1.09)
High MVI	1.39 (1.24,1.55)[a]	1.02 (0.92,1.12)
Very High MVI	1.62 (1.44,1.81)[a]	1.02 (0.92,1.13)
**Very Preterm Birth**		
Very Low MVI	Reference	Reference
Low MVI	1.07 (1.00,1.16)[b]	0.97 (0.89,1.05)
Moderate MVI	1.19 (1.10,1.28)[a]	0.97 (0.89,1.05)
High MVI	1.31 (1.21,1.41)[a]	1.01 (0.93,1.09)
Very High MVI	1.42 (1.31,1.53)[a]	0.99 (0.92,1.08)
**Moderate Preterm Birth**		
Very Low MVI	Reference	Reference
Low MVI	1.05 (0.98,1.13)	0.94 (0.86,1.02)
Moderate MVI	1.18 (1.10,1.27)[a]	0.97 (0.90,1.05)
High MVI	1.26 (1.17,1.35)[a]	0.97 (0.90,1.05)
Very High MVI	1.37 (1.26,1.49)[a]	0.99 (0.91,1.07)
**Late Preterm Birth**		
Very Low MVI	Reference	Reference
Low MVI	1.00 (0.96,1.04)	0.96 (0.92,1.00)
Moderate MVI	1.05 (1.01,1.10)[b]	0.95 (0.92,0.99)[b]
High MVI	1.12 (1.06,1.18)[b]	0.96 (0.92,1.00)
Very High MVI	1.15 (1.10,1.21)[b]	0.96 (0.92,1.00)
**Term Birth**		
	Reference	Reference

[a]$p<0.001$, [b]$p<0.05$
GA: Gestational Age; PTB: Preterm Birth
MVI is the Maternal Vulnerability Index. Very Low MVI is 0-20. Low MVI is 20-40. Moderate MVI is 40-60. High MVI is 60-80. Very High MVI is 80-100. Adjusted for sex, race, age, insurance, education, adequacy of prenatal care, nulliparous status, pre-existing hypertension, body mass index, smoking, and state.
The output from the Stata mlogit model is expressed as a ratio of relative risk which but for simplicity is reported as an odds ratio.

© 2023 Salazar EG et al. *JAMA Network Open*.

eFigure. Unadjusted and Adjusted Associations of MVI With Preterm Birth by Gestational Age Categories Compared With Term Births

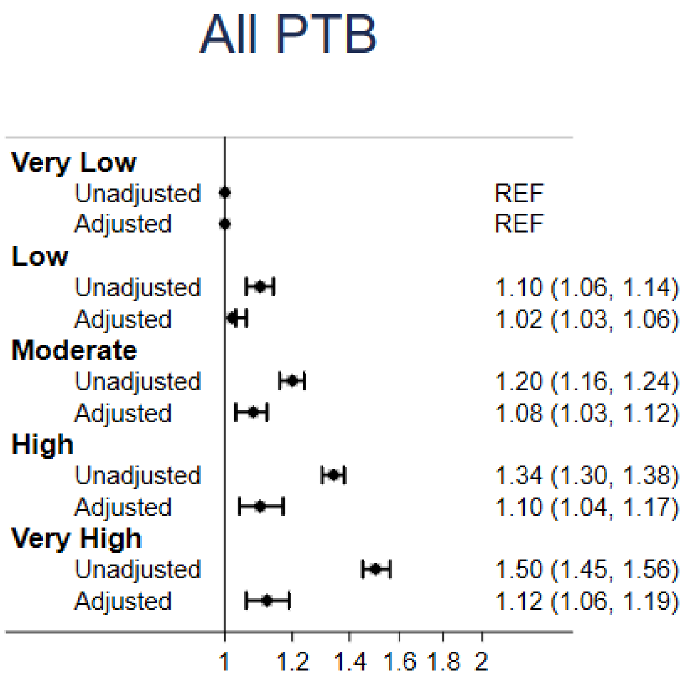

MVI: Maternal Vulnerability Index, PTB: Preterm Birth.
Adjusted for age, insurance, education, Kotelchuk index of adequate prenatal care, body mass index, nulliparity, pre-existing hypertension, smoking during pregnancy, rurality, infant sex, and census region. Associations are reported as odds ratios.

© 2023 Salazar EG et al. *JAMA Network Open*.
