# Supplementary material for: County-Level Maternal Vulnerability and Preterm Birth in the US
Source: JAMA Netw Open. 2023 May 25;6(5):e2315306. doi: 10.1001/jamanetworkopen.2023.15306 (PMC10214038; doi:10.1001/jamanetworkopen.2023.15306)
Supplement: Supplement 2. — Data Sharing Statement [file jamanetwopen-e2315306-s002.pdf]

Data Sharing Statement

Salazar. County-Level Maternal Vulnerability and Preterm Birth in the US. *JAMA Netw Open*. Published May 25, 2023. doi:10.1001/jamanetworkopen.2023.15306

Data
Data available: No

Additional Information
Explanation for why data not available: Data reported in this paper may be requested from the National Center for Health Statistics through a data use agreement.
